# Adrenocortical carcinoma in a patient with neurofibromatosis type 1: A case report

**DOI:** 10.22088/cjim.12.4.613

**Published:** 2021

**Authors:** Mohammad Najafi-Semnani, Mahdieh Rajabi-Moghaddam, Hamid Abbaszadeh

**Affiliations:** 1 Department of Urology, School of Medicine, Birjand University of Medical Sciences, Birjand, Iran; 2 Department of Pathology, School of Medicine, Birjand University of Medical Sciences, Birjand, Iran; 3 Department of Oral and Maxillofacial Pathology, School of Dentistry, Birjand University of Medical Sciences, Birjand, Iran

**Keywords:** Adrenocortical carcinoma, Neurofibromatosis 1, Adrenal glands, Adrenal cortex neoplasms

## Abstract

**Background:**

Neurofibromatosis type 1 (NF-1) is a genetic disorder. A heterogeneous group of benign and malignant neoplasms are associated with NF-1. Adrenocortical carcinoma (ACC) is an extremely rare invasive malignancy. The association of ACC with NF1 is not well understood.

**Case Presentation:**

We report a case of ACC in the context of NF1 in a 39- year-old woman who referred with the chief complaint of a mass in left abdomen. A left adrenal lesion was diagnosed by CT scan. Biochemical tests showed no abnormality. Adrenalectomy was done and histological and immunohistochemical findings confirmed ACC. Due to the absence of metastasis, clinical stage II was considered for the tumor. On follow-up after six months, she was still alive and well and with no evidence of metastasis. The age of patient and lack of secretion of adrenal cortical hormones in this case were unlike most ACCs.

**Conclusion:**

Also, modified Weiss score for malignancy of adrenocortical neoplasms, clinical staging system and different modality of treatment is discussed.

Von Recklinghausen disease or neurofibromatosis type 1 (NF-1) is a genetic disorder which occurs because of mutations of the NF1 tumor suppressor gene with an incidence of one in every 3000 to 4000 birth (1). Pathogenesis of most of the NF1 manifestations involves loss of NF1 function. Hyperactivation of the RAS pathway due to the lack of neurofibromin, the product of the NF1 gene, leads to abnormal cellular growth and proliferation and a variety of benign and malignant neoplasms (2, 3). NF1 can affect the skin, eyes, bones, veins, nerves, and a person's general constitution. The following is some of the common features associated with NF type 1: skin: Café au lait spots, dermal neurofibromas and freckling; eye: Lisch nodules; bone: sphenoid wing dysplasia; peripheral nervous system: neurofibromas and malignant peripheral nerve sheath tumor (MPNST); learning disabilities (2). Malignant tumors can arise in the nervous and non-nervous system with MPNST is the most frequent (4). NF1 has different types of manifestations within the abdomen and pelvis, some of them can mimic others and cause a diagnostic challenge (1). A non-homogenous group of benign and malignant abdominal neoplasms are associated with NF1 (2, 5). Genitourinary conditions associated with NF1 include pheochromocytoma (adrenal), neuroblastoma, Wilms tumor (kidney), serous ovarian carcinoma, testicular germ cell tumor and neurofibroma of the genital tract (reproductive system) (1). The association of pheochromocytoma with NF1 has been confirmed (6). 

The incidence of pheochromocytoma, a neuroendocrine tumor which usually develops in adrenal medulla, is approximately 0.1%–5.7% in NF1 patients (7, 8). Adrenocortical carcinoma (ACC) is an extremely rare invasive malignancy which occurs in the cortex of adrenal glands. 

The incidence is 0.7 to 2.0 cases per million per year (9). The diagnosis of this condition is challenging. ACC has seldom occurred in NF1 patients (6). There is no definite association between NF1 with ACC (6, 10).

## Case Presentation

**Figure 1:**
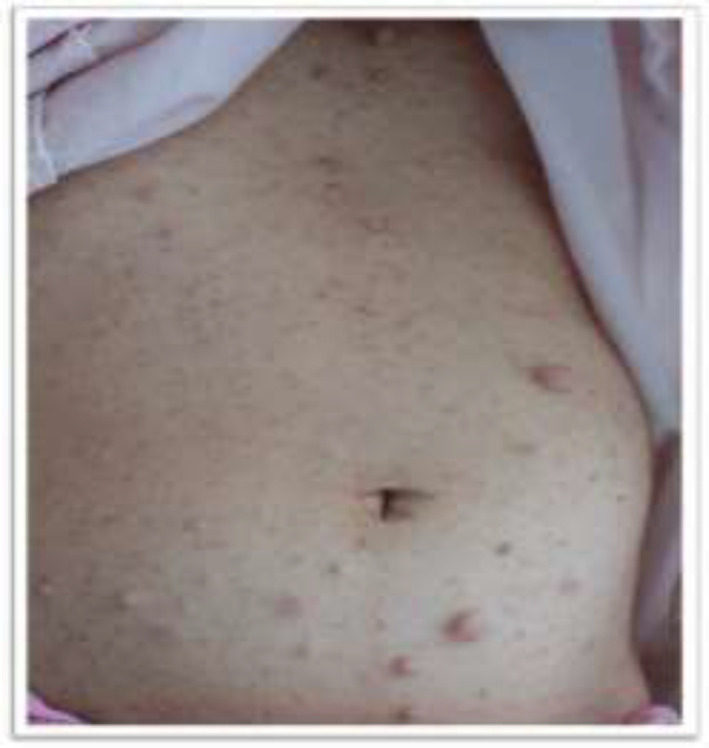
Multiple cutaneous neurofibromas on skin of hands and abdomen

This report was confirmed by ethics committee of our university (# IR.BUMS.REC.1399.545). Conscious consent was obtained from the participant. A mentally retarded 39-year-old female referred to urology unit of Razi Hospital in Birjand, Iran, with a painless lesion on left side of her abdomen for one month duration. There were brown spots and soft tissue masses throughout her body from childhood. Past medical history showed that the patient was a confirmed case of neurofibromatosis type 1, diagnosed at the age of 8; she had multiple Café-au-lait skin lesions from that time and gradually developed learning disabilities. She was oriented during examination. There was no family history of endocrine malignancy or endocrinopathy. The cranial nerve examination was normal. Blood pressure was 105/65 mmHg. Café-au-lait macules, axillary freckling and multiple masses compatible with cutaneous neurofibromas were evident on skin (figure 1). 

**Figure 2:**
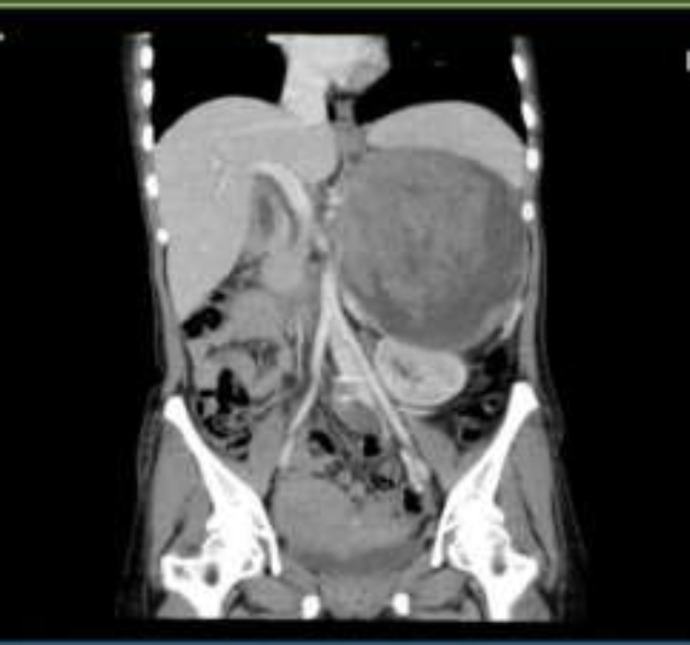
Abdominal and pelvic CT scan showing a heterogeneous mass with in the left adrenal anatomical location in the vicinity of the tail of the pancreas

On ocular examination, bilateral peripapillary atrophy, optic disc pallor and Lisch nodules were evident. Abdominal and pelvic CT scans and MRI were requested for the patient. On CT scan, a heterogeneous mass with internal necrotic areas and small foci of calcification in the vicinity of the tail of the pancreas was observed; its dimensions were 152 137 110 mm; its boundaries were not distinguished from the pancreas. On MRI, a heterogeneous area with the dimensions of 146 113 mm in the left adrenal was reported; it included cystic areas and internal vessels and also had the compressive effect on the right kidney (figure 2). 

**Figure 3:**
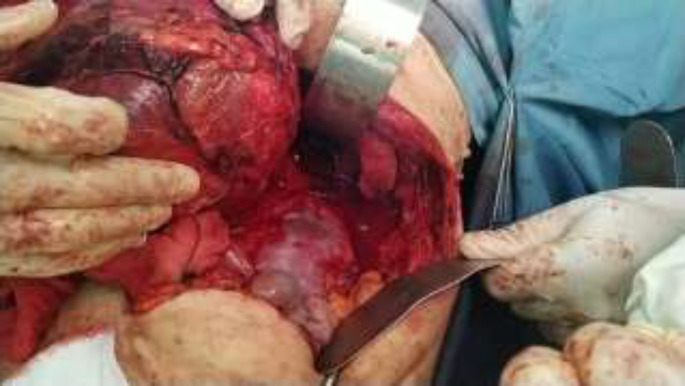
Intraoperative view of complete tumor resection

Additional biochemical tests were requested with suspicious to adrenal neoplasms especially pheochromocytoma. Urine analysis showed that normetanephrine was 291 μg/24 hours (reference value: <600 μg/24 hours) and vanillylmandelic acid (VMA) was 4.3mg/24 hours (reference value in adult: up to 13.6 mg/24 hours). Cortisol AM was 18.1μg/dL (reference value in 8AM-10AM: 3.95-27.2). Dehydroepiandrosterone sulfate (DHEAS) was 0.38 μg/ml (reference value in females: 0.35-4.2). These findings were not compatible with the clinical diagnosis of pheochromocytoma. The patient became a candidate for surgical removal of tumor. The patient underwent surgery with thoracoabdominal approach on the tenth rib. There was so much bleeding during the operation. There was severe tumor adhesion to the diaphragm and retroperitoneum around the aorta and superior vena cava. The tumor was completely excised with intact capsule. Lymphadenectomy was done for aortic lymph nodes (figure 3).

**Figure 4:**
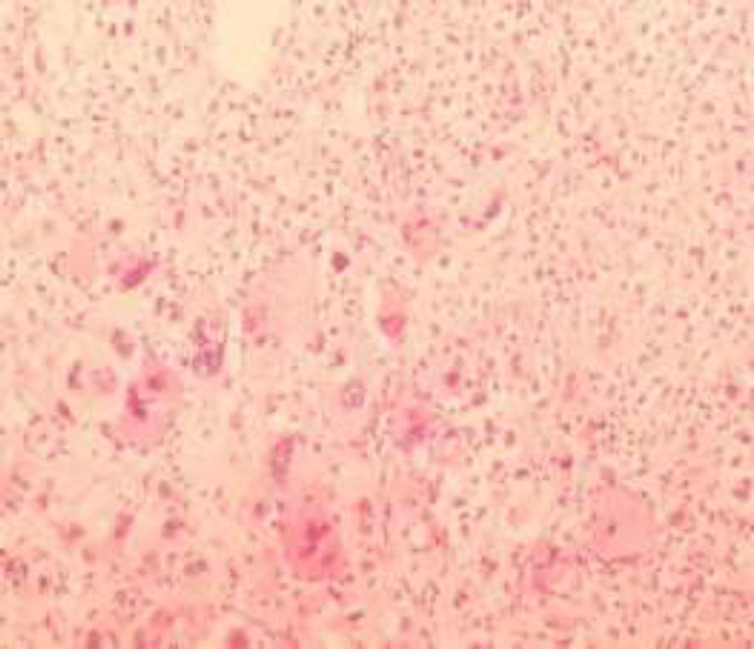
Presence of giant cells with bizarre pleomorphic nuclei on microscopic examination

The patient was discharged from the hospital 7 days after the operation. The tumor was sent for histopathological examination. On microscopic examination, an encapsulated neoplastic proliferation with a diffuse pattern was seen composed of giant cells with abundant acidophilic cytoplasm and bizarre pleomorphic and vesicular nuclei, occasionally with prominent nucleoli and intranuclear halo. Atypical mitotic figures, hemorrhage, necrosis and inflammatory infiltration were also noted. Remnant of normal adrenal tissue was seen. Occasionally, tumor cells had a perivascular pattern (figure 4). 

Lymph nodes showed no evidence of neoplastic cells. Microscopic findings were suggestive of a malignant neoplasm with the following differential diagnosis: 1- Adrenocortical neoplasm 2- Adrenomedullary neoplasm 3- Metastatic tumor. For differential diagnosis immunohistochemical (IHC) studies for chromogranin, synaptophysin (neuroendocrine markers), Melan-A, vimentin, CD-10, Inhibin, and Cytokeratin AE1/AE3 were recommended. The cells of tumor stained for synaptophysin and did not stain for Pax8, cytokeratin AE1/AE3, CD10, EMA and Chromogranin; the tumor cells were focally stained for Melan-A, vimentin and Inhibin. Based on histopathological and immunohistochemical findings, the diagnosis of adrenocortical neoplasm was confirmed. According to Weiss criteria, modified for considering an adrenocortical tumor as malignant (11), findings were compatible with adrenocortical carcinoma. A chest CT scan and whole-body bone scan was performed and no suspected mass was found on the scans. Due to the absence of regional and distant metastases, the clinical stage II was assigned to the tumor. Six months after the diagnosis of the tumor, the patient was still alive and well; no particular problems were reported by the patient and her family. The patient did not show manifestation of inadequate production of adrenal hormones and biochemical findings were also normal. Follow-up CT scans did not find any evidence of metastasis.

## Discussion

Ten to fifteen percent of ACCs are inherited with a mutated gene but there is no evidence to suggest an association between NF-1 and ACCs (10). ACCs are very rare malignancies and seen with a greater frequency in children less than 5 years old and adults over 40 years of age; two thirds of them are capable of secreting an excess of corticosteroid hormones (hormonally functional) which can result in malignant hypertension, Cushing disease and virilization; nevertheless, non-functional tumors are occasionally detected. However, non-functional tumors cause a diagnostic challenge because they are diagnosed incidentally (10). In our case, the patient's age did not match with the age distribution of patients of ACC. Due to the lack of secretion of corticosteroid hormones, in our case, this was not consistent with that in the majority of ACCs; malignant hypertension, Cushing disease and virilization were not observed in our case. 

Women affected more frequently to ACCs; functional ACCs are seen more frequently in women (11). In terms of gender, our case is consistent with most ACCs, but the cancer in our case, despite occurring in a woman, was non-functional.

Weiss criteria, modified for considering an adrenocortical tumor as malignant are: Mitoses >5 per 50 HPFs; atypical mitoses; clear cells ≤ 25% of tumor cells; necrosis, capsule invasion. In this system, calculation is as follows: 2 mitoses + 2 clear cytoplasm + atypical mitoses + necrosis + capsule invasion; score is 0 for criteria that do not exist and score is 1 for criteria that exist; score ≥ 3 is in favor of a malignant neoplasm (11). The histopathological findings of our case met all Weiss criteria except capsular invasion. As a result, modified Weiss score was 6; this score is in favor of malignancy (adrenocortical carcinoma).

Kollurage et al. reported a case of metastatic ACC in a five-month-old baby with NF1 which presented with adrenal crisis (10); the adrenal crisis in their case was contradictory to our case. Most of adrenocortical carcinomas metastasized at onset (10). There was a difference between our case and the case report of Kollurage et al. and the majority of ACCs due to the absence of metastasis at the time of diagnosis.

Menon et al. presented a 49 -year-old woman who had adrenocortical carcinoma along with NF-1 (6). Laboratory reports indicated mild increase in production of cortisol and increased amounts of androgens. They reported elevated levels of androgen precursors including DHEA as well as the glucocorticoid precursor. These biochemical findings were contradictory to those in our case. This is because the ACC in their case was functional and produced cortisol, but in our case, it was non-functional and did not produce hormones. Clinically, their case was not in favor of pheochromocytoma. The clinical manifestations of both cases were similar in this respect. The size of tumor in their case report was 8.7 8.6 cm. The size of the tumor of our case was about twice the size of the tumor of their case. There was coordination between the two case reports with respect to absence of metastasis. As in our case, they chose open adrenalectomy to treat their patient. Modified Weiss scores of both cases were 6. Clinical stage of both cases was the same.

Wagner et al. reported a 3-year-old girl with NF1 who showed virilization; excision of adrenal gland showed an adrenocortical carcinoma which metastasized after a few months (12); Virilization and systemic metastases in their case were in contradictory to our case. After a post-surgical course, adjunctive chemotherapy was done. In their case, unlike in our case, the patient underwent chemotherapy due to metastasis.

Gutmann et al. reported an adrenal gland tumor from a 49-year-old female with neurofibromatosis type I (13); it is not clear whether this was an ACC. Minkiewicz et al. presented a 57-year-old female with NF-1 who presented during her lifetime three neoplasms including endometrial cancer, ACC and gastrointestinal stromal tumor (GIST) (14). Unlike our case, their case had hypertension at diagnosis. Similar to our case, cortisol, androgens, and catecholamine metabolite levels were within the normal range. IHC report of their case was as follows: Melan A (+), inhibin (-), chromogranin A (-), synaptophysin (-), S100 (-). Except synaptophysin, the other findings were the same as in our case.

European Network for the Study of Adrenal Tumors (ENSAT) stage is used for staging of adrenal cancers. In this system, stage I means T1 N0 M0, stage II is T2 N0 M0, stage III is T1–2 N1 M0 or T3–4 N0–1 M0 and stage IV means T any N any M1 (15). Our case was T2N0M0. According to this system, stages II tumor was assigned to this tumor.

Treatment of ACC depends on the tumor spread, general well-being and fitness of the patients for combined surgery, chemotherapy and radiotherapy (10). In non-metastatic ACC (stage I to stage III), surgical excision to resect the tumor completely is the optional treatment. Recurrence is common if the tumor invades the surrounding capsule (16). Surgery has not been recommended in stage IV ACC (metastatic ACC) (15). If you decide to resect the tumor in stage IV ACCs, it should be considered personally. Patients with metastasis to several distant organs or patients who had several metastatic masses in one organ which is not possible to completely remove them with surgery are not candidates for adrenalectomy. In these cases, the tumor is managed by radiotherapy accompanied with other adjunctive therapy. Mitotane is the adjunctive treatment in such a case (16).

The prognosis of patients with ACC is poor and the five-year survival rate is between 16 to 35% (16).

Early diagnosis of abdominal manifestations in NF1 patients is very important given the risk of malignancy; it is important to evaluate these patients annually in a specialized center consisting of multidisciplinary physicians to allow the early detection of complications and formation of neoplasms.
